# Newer Insulin Preparations and Insulin Analogs

**DOI:** 10.7759/cureus.74593

**Published:** 2024-11-27

**Authors:** Devkumar D Tiwari, Vandana M Thorat, Dr.Prathamesh V Pakale

**Affiliations:** 1 Department of Pharmacology, Krishna Vishwa Vidyapeeth (Deemed to be University), Karad, IND

**Keywords:** diabetes mellitus type i, frederick banting, long-acting insulin, porcine, reference insulin glargine

## Abstract

Diabetes mellitus represents a significant and growing global health challenge, with its prevalence steadily increasing. Insulin therapy remains a cornerstone of diabetes management. Since its discovery in 1921, insulin has undergone substantial advancements, evolving from crude animal extracts to highly refined recombinant formulations and biosimilars. This review explores the progression of insulin therapies, emphasizing the evolution from conventional insulins to modern analogs designed to mimic endogenous insulin more effectively.

The limitations of early insulin formulations, such as unpredictable absorption, rigid dosing regimens, and an increased risk of hypoglycemia, highlighted the need for improved therapies. Modern insulin analogs, including fast-acting (e.g., insulin lispro), long-acting (e.g., insulin glargine and insulin degludec), and ultra-long-acting (e.g., insulin icodec) options, address these challenges by providing stable and consistent pharmacokinetics, along with enhanced glycemic control. Furthermore, biosimilar insulins, produced via recombinant DNA technology, have increased accessibility while maintaining therapeutic efficacy and safety.

Recent innovations, such as ultra-long-acting insulins and combination therapies like insulin icodec with semaglutide, offer the potential to reduce injection frequency and enable personalized diabetes care. These advancements contribute to improved patient compliance, reduced glycemic variability, and an enhanced quality of life. This review highlights the critical role of ongoing research and innovation in insulin therapy to meet the evolving needs of diabetes management.

## Introduction and background

Diabetes is a chronic metabolic disease with a high global prevalence, rapidly reaching epidemic proportions. In 2019, it affected 9.3% of the world's population, approximately 463 million people. The global prevalence of diabetes is expected to rise to 10.2% (578 million people) by 2030 and 10.9% (700 million people) by 2045 [1]. All patients with type 1 diabetes mellitus (T1DM) require insulin due to its absolute deficiency. As life expectancy increases among patients with type 2 diabetes mellitus (T2DM), they too will require insulin due to progressive β-cell failure [2].

The discovery of insulin over a century ago remains one of the most significant medical advancements of the 20th century. The production of bovine and porcine insulin began shortly after Banting and Best's discovery in 1921. While early insulin preparations contained a significant amount of contaminants, advancements in manufacturing processes have greatly improved their safety, leading to the development of safer bovine and porcine insulin preparations. The first successful insulin treatment was administered on January 11, 1922, using an insulin extract known as "Macleod serum" [3]. In the 1930s, Danish chemist HC Hagedorn added protamine to insulin, and later, Scott and Fisher in Toronto added zinc to further extend insulin's effects. Protamine zinc insulin had a duration of 24-36 hours. Isophane or neutral protamine Hagedorn (NPH) insulin, one of the first longer-acting insulins, had a half-life of 24 hours. Eli Lilly and Company began producing insulin in large volumes in 1936. Insulin was the first human protein to be sequenced and synthesized using human recombinant DNA technology in 1955. Slower-acting insulins were also discovered, with Novo Nordisk Inc. being the first to introduce them. The first synthetic "human" insulin, genetically modified using *Escherichia coli*, was produced in 1978. In 1982, the first commercially available synthesized human insulin, marketed under the name Humulin, was introduced [2,4,5].

These accomplishments signaled a halt in insulin product development until 1996, when the results of the Diabetic Control and Complication Trial (DCCT) and the United Kingdom Prospective Diabetic Study (UKPDS) were published, confirming the important role of glycemic control in preventing or delaying diabetes complications [6,7]. Due to the limited pharmacokinetic and pharmacodynamic characteristics of standard insulins, which frequently result in hypoglycemia as glycosylated hemoglobin levels approach the normal range, there has been renewed interest in developing safer insulin formulations that more closely mimic the basal and mealtime components of endogenous insulin secretion. This has led to the development of insulin analogs with action profiles that enable more flexible treatment regimens and a reduced risk of hypoglycemia [8].

## Review

Insulin

The development of insulin has a rich history spanning more than a century, beginning with the discovery of pancreatic cells. Since then, insulin has evolved from an extracted hormone to sophisticated synthetic analog formulations and regenerative therapies, revolutionizing diabetes management and improving patient outcomes marked by significant milestones in biomedical research and therapeutic innovation.

This structured timeline progression captures and reflects key breakthroughs and significant biomedical advancements, illustrating the transformation of diabetes management from basic hormone replacement to advanced, life-enhancing treatments. From insulin’s initial extraction in the early 20th century to today's advancements in gene programming and stem cell research, each milestone marks a pivotal moment that has reshaped diabetes treatment, improving the quality of life and survival for millions worldwide [9-20].

History

The history of insulin preparations and analogs dates back to the early 1920s when insulin was first discovered by Frederick Banting and Charles Best at the University of Toronto. In 1922, insulin was successfully used for the treatment of diabetes, with initial preparations being derived from animal sources, primarily bovine and porcine pancreas. During the 1950s and 1960s, insulin was further purified and standardized, and by the 1970s, recombinant DNA technology allowed for the production of human insulin, marking a significant advancement in diabetes care. In 1982, recombinant human insulin (Humulin), produced using genetic engineering, became the first commercially available synthetic insulin. The 1990s saw the introduction of insulin analogs, such as insulin lispro (Humalog) in 1996, which is a rapid-acting insulin analog designed to more closely mimic the body’s natural insulin response. This was followed by the approval of insulin glargine (Lantus) in 1999, a long-acting insulin analog that provides stable, prolonged insulin action with fewer fluctuations in blood sugar levels.

**Figure 1:**
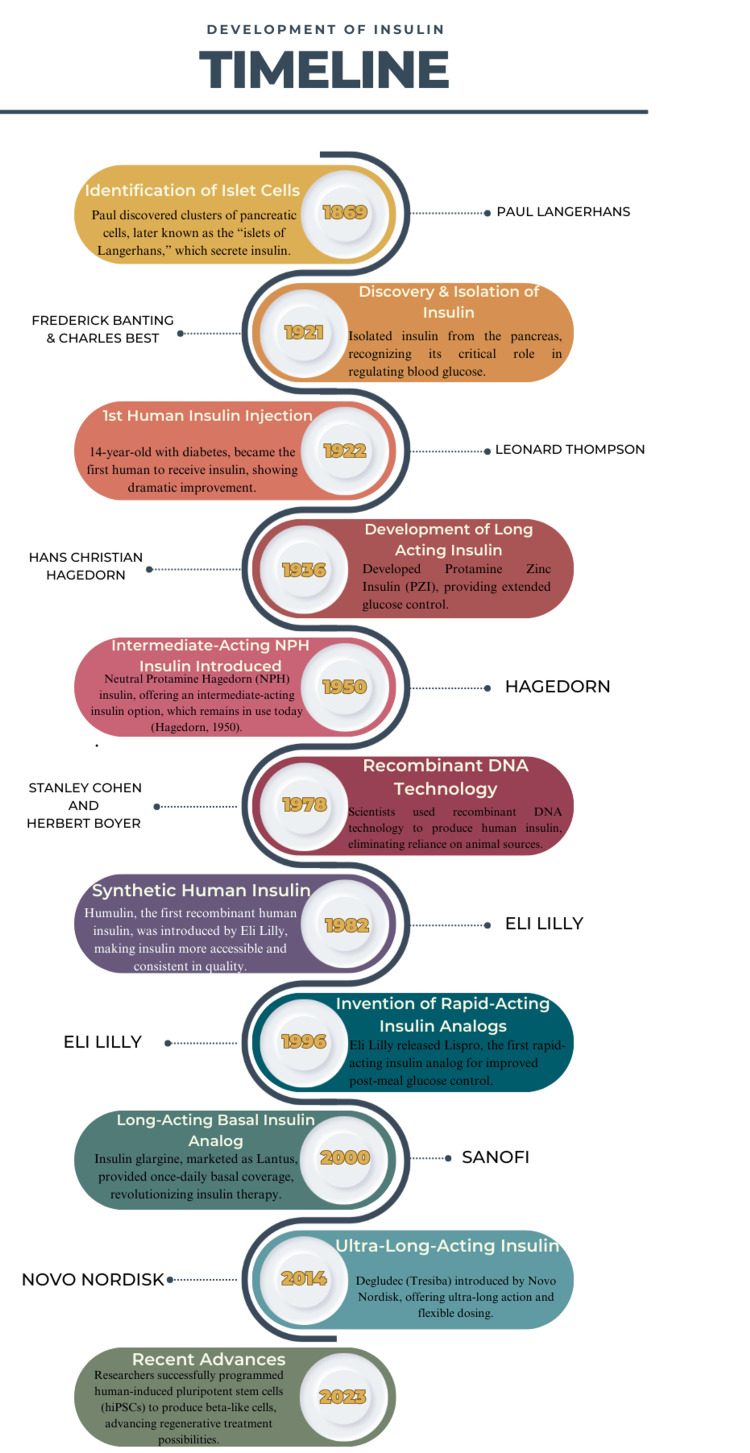
Timeline of the development of insulin This image was illustrated by Mr. Devkumar D. Tiwari, one of the authors.

In the 2000s, rapid-acting insulins like insulin aspart (NovoRapid) and long-acting analogs like insulin detemir (Levemir) were introduced, providing more flexible and precise options for managing blood glucose. The 2010s witnessed the approval of insulin degludec (Tresiba) in 2014, an ultra-long-acting insulin with a duration of action of up to 42 hours, offering even more flexibility in diabetes management. Additionally, faster-acting insulin formulations such as Fiasp, introduced in 2019, were developed to act more quickly post-injection, improving postprandial glucose control. Research continues into even faster-acting insulins, insulin inhalers, and new insulin delivery systems such as insulin pumps and closed-loop systems that integrate insulin delivery with continuous glucose monitoring to offer personalized, real-time diabetes management. The ongoing evolution of insulin preparations reflects a shift toward greater convenience, efficacy, and safety, with an emphasis on reducing the risk of hypoglycemia and providing more precise control over blood sugar levels (Figure 1).

Advantages of newer insulins and disadvantages of conventional insulins

Conventional insulin therapies, primarily derived from animal sources or early synthetic formulations, were groundbreaking at their time but came with several limitations [8]. Over time, advancements in biotechnology led to the development of newer insulin analogs, offering more predictable pharmacokinetics and better alignment with the body's natural insulin response. Advancements in biotechnology have significantly transformed insulin therapy, leading to the development of insulin analogs that closely align with the body's natural insulin response. The journey began with recombinant DNA technology, which allowed for the synthesis of human insulin by inserting the insulin gene into bacterial or yeast cells, enabling large-scale production of insulin identical to human insulin. Building on this foundation, structural modifications to the insulin molecule resulted in analogs with enhanced pharmacokinetic properties. Rapid-acting analogs, such as insulin lispro, aspart, and glulisine, were designed to act swiftly post-injection, addressing postprandial glucose spikes effectively. Conversely, long-acting analogs like insulin glargine and degludec offer a stable, prolonged effect, mimicking basal insulin secretion and reducing the risk of nocturnal hypoglycemia. These structural changes also optimized absorption rates, solubility, and stability. For instance, insulin degludec forms multi-hexamer complexes that dissolve slowly, ensuring ultra-long action. Additionally, the introduction of biosimilar insulins, produced via advanced recombinant techniques, has improved accessibility without compromising safety or efficacy. Innovations have further extended to fixed-ratio combination therapies, such as insulin analogs paired with GLP-1 receptor agonists, which offer synergistic benefits for better glycemic control. Collectively, these advancements provide more predictable pharmacokinetics, enhanced glycemic regulation, and reduced side effects, greatly improving diabetes management and patient quality of life [21]. These innovations have not only improved glucose management but also enhanced the quality of life of individuals with diabetes, making insulin therapy safer and more effective [22].

**Figure 2:**
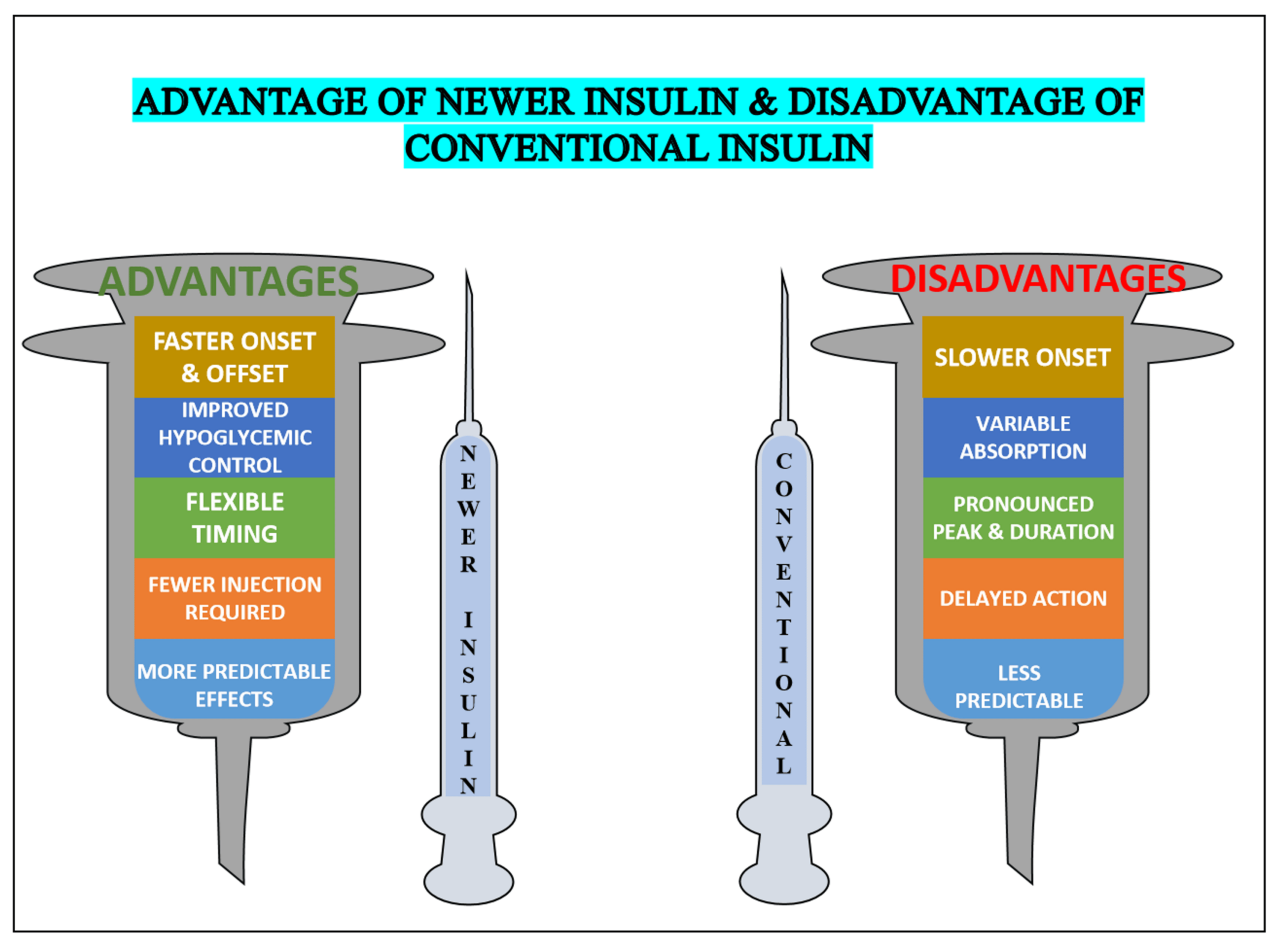
Advantages of newer insulin and disadvantages of conventional insulin This image was illustrated by Mr. Devkumar D. Tiwari, one of the authors.

Figure 2 shows the advantages of newer insulins and the disadvantages of conventional insulins.

The following points highlight the limitations of conventional insulin in achieving optimal glucose control and patient convenience, as identified in published studies.

Unpredictable Absorption

Conventional insulin has variable absorption rates, leading to inconsistent glucose control [23].

Risk of Hypoglycemia

The delayed or prolonged action of conventional insulin increases the risk of hypoglycemia, especially overnight or between meals [8].

Peak Timing Issues

Peaks in insulin action do not always align with blood sugar spikes, requiring precise meal timing, which limits flexibility [17].

Immunogenicity

Animal-derived insulin can trigger immune responses, leading to allergic reactions or reduced insulin effectiveness over time [21].

Rigid Dosing Schedules

Multiple injections at fixed times restrict patients' ability to adapt insulin administration to their schedules [22].

Short Duration of Action

Some conventional insulin does not provide full 24-hour coverage, increasing the need for additional doses or mealtime adjustments [16].

Allergic Reactions

Impurities in animal-sourced insulin cause allergic reactions in some patients, complicating the therapy [24].

Increased Dose Requirement

Immunogenic reactions can result in higher antibody levels, necessitating larger insulin doses [21].

Higher Risk of Weight Gain

The use of Conventional insulin often leads to weight gain due to frequent adjustments to manage peaks and lows [7].

Lack of Flexibility

Conventional insulin requires strict adherence to timing, reducing patients' ability to manage diabetes in their daily lives [22].

On the other hand, the following points highlight the advantages of newer insulin in achieving optimal glucose control and patient convenience, as identified in published studies. 

Predictable Pharmacokinetics

Newer insulin analogs offer consistent absorption and predictable action times, enhancing glucose control [25].

Reduced Hypoglycemia Risk

Long-acting analogs like glargine and degludec minimize unexpected drops in blood sugar, especially overnight [21].

Flexible Dosing

Analog insulins provide flexible dosing schedules, allowing patients more freedom in meal timing [8].

Rapid Response Post-Meal

Rapid-acting analogs, such as Lispro, work quickly to control post-meal glucose levels effectively [26].

Lower Immunogenicity

Recombinant DNA production reduces allergic reactions and immune responses, improving insulin tolerance [16].

Improved Quality of Life

Fewer hypoglycemic events and flexible dosing contribute to reduced anxiety and better daily functioning [22].

Longer-Acting Coverage

Ultra-long-acting insulin provides stable basal control for up to 42 hours, reducing injection frequency [19].

Reduced Insulin Antibody Formation

Purity in modern analogs prevents the need for increased doses, maintaining efficacy over time [27].

Convenient Administration

New analogs support less frequent injections and flexible meal timings, enhancing patient convenience [28].

Enhanced Nighttime Control

Steady basal insulin analogs help prevent nighttime hypoglycemia, promoting safer overnight glucose management [29].

Newer insulin

In 1996, genetic engineering enabled the development of novel long- and short-acting insulin analogs, which cleared the path for more physiological insulin therapy in terms of hypoglycemia and patient satisfaction. They were potentially less problematic and made the treatment more adaptable, safer, easier, cheaper, and straightforward. Faster-acting pre-prandial insulin and longer-acting basal insulin are examples of newer insulins that deliver a steady concentration with no peak spike in insulin levels. Newer analogs occur as monomers and are either rapidly absorbed or slowly absorbed. The newer analogs seem to be more stable, have less complexity, and provide a more specific impact, which aid in the development of customized treatments tailored to specific patient parameters and provide better glycemic control [30,31].

**Figure 3:**
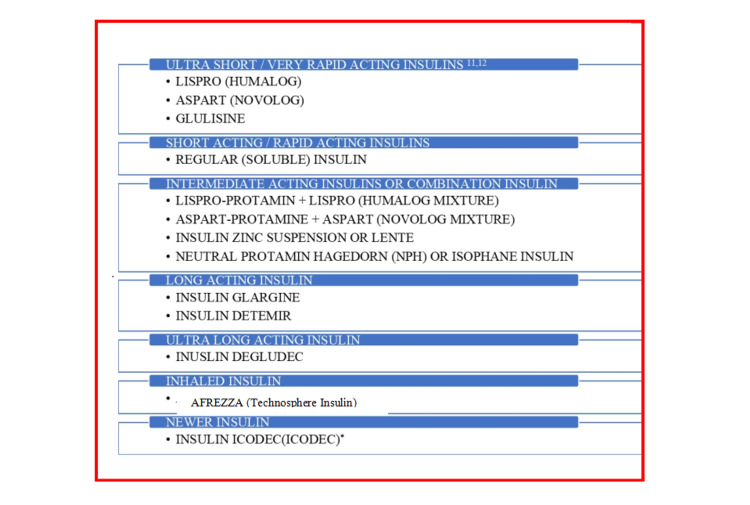
Insulin analogs Source: [3,33-41].

Biosimilars and interchangeable biologics, which are "generic" biologics, were approved by the FDA in 2010. Generic medications undergo a similar procedure. In a significant development, insulin was moved to a biologic regulatory framework on March 23, 2020. This change implies that the FDA has officially classified all insulins on the market as biologics, paving the path for biosimilar and interchangeable insulins (Figure 3) [32].

Types of insulin preparations

Banting and Best discovered insulin in 1921 when they showed that a pancreatic extract, obtained after the exocrine portion of the organ degenerated due to pancreatic duct ligation, had hypoglycemic effects. It was initially discovered in pure crystal form in 1926, and Sanger completed its chemical structure in 1956 [42].

Insulins made from beef and pork are no longer produced in the USA, but they are still sold in the UK, India, and a few European nations. More than 90% of diabetics in Britain currently use human insulins or insulin analogs, oral antidiabetic medications, and glucagon 287. Apart from financial concerns, human insulins and their analogs are also widely used in India. Compared to pig or cow insulin, the human insulin is more hydrophobic and water-soluble. It has a significantly shorter duration of action, an earlier and more distinct peak concentration, and a slightly faster subcutaneous absorption [43].

**Figure 4:**
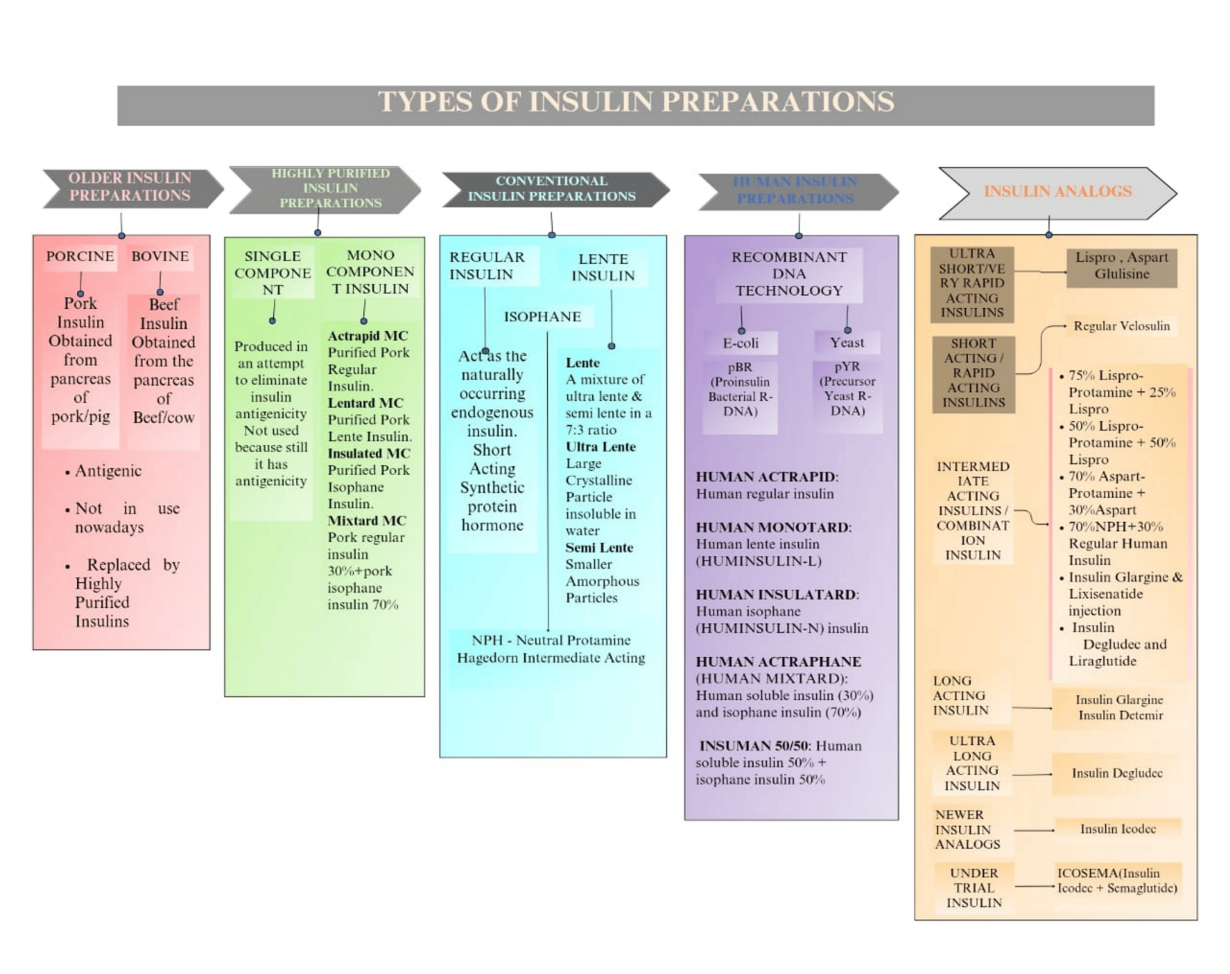
Classifications of insulin preparations and analogs This image was illustrated by Mr. Devkumar D. Tiwari, one of the authors.

The following text describes the classifications of insulin preparations and analogs, as shown in Figure 4.

Older Insulin Preparations

In the past, the pancreas from cattle and pork were used to make insulin formulations. Proinsulin, other polypeptides, pancreatic proteins, insulin derivatives, and other proteins that might be antigenic were present in them in amounts of about 1% (10,000 ppm). These insulins are no longer made and have been completely superseded by highly pure insulins from beef or pork, human recombinant insulins, and insulin mimics [43].

Highly Purified Insulin Preparations

In the 1970s, "single peak" and "monocomponent (MC)" insulins with a concentration of not more than 10 ppm proinsulin were created using advanced purification methods such as gel filtration and ion-exchange chromatography.

MC insulins: Insulin resistance and injection site lipodystrophy are less common with the more stable MC insulins. Recombinant human insulin and pig MC insulin both have comparable immunogenicity.

Single-component (SC) insulins: They were produced in an attempt to eliminate insulin antigenicity. However, because they still have antigenicity, they are not used [44].

Conventional Insulin Preparations

Regular (soluble) insulin: It is an unaltered insulin buffered with a pH-neutral solution, which has been slightly stabilized with a minute amount of zinc. It is optimally injected one hour before a meal. Subcutaneously injected insulin is also unsuitable for maintaining a low, constant basal level of action during the inter-digestive period. However, the slow onset of action does not apply to intravenous injection, because the insulin hexamer dissociates rapidly to produce prompt action. The only insulin used for intravenous injection is regular insulin.

Lente insulin (insulin-zinc suspension): There are two varieties of insulin-zinc suspensions available. The large-particle variety is crystalline and nearly water-insoluble (ultralente). It has a long half-life. The other variety is short-acting, is amorphous (semilente), and contains smaller particles. "Lente insulin" refers to their 7:3 ratio combination, which provides an intermediate-acting effect.

Isophane (NPH) insulin: There is no free form of either insulin or protamine, and the pH is neutral. Protamine is added in sufficient amounts to complex all insulin molecules. It is mostly combined with regular insulin (70:30 or 50:50) and injected subcutaneously twice daily, before breakfast and before dinner, as part of a split-mixed regimen.

*Human Insulin Preparations*****

Human insulins with the same amino acid sequence were created in the 1980s through two different methods using recombinant DNA technology (enzymatic modification of porcine insulin (emp) and proinsulin recombinant bacterial (prb) in *Escherichia coli* and precursor yeast recombinant (pyr) in yeast) [14,45]: Human Actrapid [45] (human regular insulin); Human Monotard [46], Huminsulin L (human lente insulin; 40 U/mL, 100 U/mL); Human Insulatard [47], Huminsulin N (human isophane insulin; 40 U/mL); Wosulin-N (40 U/mL inj. vial and 100 U/mL pen injector cartridge); Human Actraphane [48], Huminsulin 30/70, Human Mixtard (human soluble insulin (30%) and isophane insulin (70%); 40 U/mL and 100 U/mL vials); Insuman 50/50 [49] (human soluble insulin 50% + isophane insulin 50% (40 U/mL inj)); Huminsulin 50:50, Human Mixtard 50; Wosulin 50/50 (40 U/mL vial, 100 U/mL cartridge).

Insulin Analogs

Analogs of insulin featuring modified pharmacokinetics following subcutaneous administration but comparable pharmacodynamic effects and immunogenicity have been created through the use of recombinant DNA technology. The additional advantages incorporate increased uniformity and stability of the preparations.

Ultra-short-acting/very rapid-acting insulin: An example would be insulin lispro. Proline and lysine are reversed at the carboxyl terminus B28 and B29 to produce insulin lispro, which forms very weak hexamers that dissociate quickly after subcutaneous injection, giving it a shorter duration of action and a quicker, more defined peak. Individual variability in absorption is minimized. Unlike regular insulin, it is best injected subcutaneously 0-20 minutes before a meal. A decreased incidence of late postprandial hypoglycemia and an improved management of mealtime glycemia have been achieved. Insulin lispro injections administered two or three times a day at mealtime have been shown to reduce HbA1c significantly in comparison to regular insulin. There were fewer occurrences of hypoglycemia (Humalog 10-mL vial, 3-mL cartridge, 100 U/mL) [50]. Another example is insulin aspart. Aspartic acid takes the role of proline at position B28 in human insulin. This alteration reduces the potential for self-aggregation and produces a time-action profile resembling insulin lispro. It has the same benefits as above and more closely resembles the typical pattern of insulin release following a meal (Novolog, Novorapid 100 U/mL injection). The 70:30 mixture of isophane complex of insulin aspart with uncomplexed insulin aspart has the advantage of rapid and predictable onset along with intermediate duration of action. It is called ‘biphasic insulin aspart’ and can be injected twice daily just before each major meal (NovoMix 30 FlexPen 100 U/mL in 3 mL injection, also as Penfill injection) (Figure 4) [51]. One more example is insulin glulisine. Another extremely fast-acting insulin analog, this one substitutes glutamic acid for lysine at B29 and lysine for asparagine at B23. The benefits and characteristics are comparable to those of insulin lispro. It has been particularly used for continuous subcutaneous insulin infusion (CSII) by a pump [52].

Short-acting/rapid-acting insulin: Examples would be regular (soluble) insulin [53] and all preparations except insulin glargine.

Intermediate-acting/combination insulin: Examples would be insulin zinc suspension or lente [54] and regular NPH or isophane insulin [55].

Long-acting insulin: An example would be insulin glargine. Glycine takes the place of asparagine at A21 in this long-acting biosynthetic insulin, which also includes two extra arginine residues at the carboxyl terminus of the B chain. At pH 4 of the formulation, this counterpart is still soluble, but upon subcutaneous injection, it precipitates at neutral pH. Monomeric insulin progressively dissociates from the depot to enter into the circulatory system of the body. Relatively low blood levels of insulin are maintained for up to 24 hours; however, the onset of effect is delayed. The final outcome is a smooth "peakless" appearance. In order to provide background insulin activity, it is appropriate for once-daily injection. No matter the time of day or the location of the subcutaneous injection, it efficiently lowers fasting and inter-digestive blood glucose levels. Most often, it is administered right before bedtime. Compared to isophane insulin, a lower frequency of hypoglycemic episodes during the night has been documented. It is unable to regulate glycemia during meals, which is treated with an oral hypoglycemic or rapid-acting insulin. It must be injected separately because It cannot be combined with any other insulin preparation due to its acidic pH [56].​ Another example is insulin detemir. Myristoyl (fatty acid) radical is attached to the amino group of lysine at B29 of the insulin chain. As a result, it binds to albumin after subcutaneous injection from which the free form becomes slowly available. A pattern of insulin action almost similar to that of insulin glargine is obtained, but twice-daily dosing may be needed [57].

Ultra-long-acting insulin: An example would be insulin degludec.​​ It is a new ultra-long-acting insulin analog with a flat plasma glucose-lowering effect lasting for ~40 hours, suitable for meeting basal insulin requirements in patients with type 1 and type 2 diabetes. After single-daily injections, the day-to-day variability in response and the risk of nocturnal hypoglycemia are lower compared to insulin glargine. An alternate-day regimen has also been tested, but may not be satisfactory. It has also been co-formulated with rapid-acting insulin aspart [58].

Newer insulin analogs:​​ An example would be​​​​​ insulin icodec. Insulin icodec is a novel once-weekly basal insulin analog [59].

Under-trial insulin: This is a combination of insulin icodec and insulin semaglutide (ICOSEMA) [60].

## Conclusions

The landscape of diabetes management has come a long way, thanks to the continuous evolution of insulin preparations and analogs. What was once a rigid and cumbersome treatment is now more flexible, personalized, and patient-friendly. From the early days of animal-derived insulin to today’s sophisticated, biosynthetic, and recombinant human insulins, these advancements have dramatically improved how insulin works in the body. Modern insulin therapies are more predictable, with consistent absorption rates and better alignment with our natural insulin production, making glucose control more stable and reliable. While conventional insulin treatments like regular insulin and NBPH were groundbreaking at the time of their introduction, they often posed challenges, including unpredictable absorption, an increased risk of hypoglycemia, and less flexibility in dosing schedules. But the newer insulin analogs, such as rapid-acting insulins like lispro and aspart, as well as long-acting options like glargine and detemir, have helped overcome many of these issues. These newer options offer patients better control of their blood glucose levels, greater safety, and much-needed flexibility, reducing the frequency of injections and providing more freedom around meal timing. Looking ahead, the development of ultra-long-acting insulins like insulin icodec, which could offer once-weekly injections, along with combinations such as insulin icodec with semaglutide, offers exciting possibilities for simplifying diabetes care even further. These innovations promise more tailored treatment plans, which could make managing diabetes less of a daily burden for patients.

As insulin therapy continues to evolve, ongoing research into newer analogs and their combinations will likely lead to even better options, improving both patient outcomes and quality of life. The growing role of biosimilars and interchangeable insulins also holds promise for making these therapies more accessible and affordable worldwide, helping ensure that more people benefit from these breakthroughs. Ultimately, the continued refinement of insulin therapies is crucial in tackling the global rise in diabetes and its associated complications, offering hope for a future where managing diabetes is easier, safer, and more effective.
